# Supplementary material for: Integrated analysis of the aqueous humor microbiome and lens capsule transcriptome in high myopia cataract: a pilot study
Source: Front Med (Lausanne). 2026 Jun 16;13:1845205. doi: 10.3389/fmed.2026.1845205 (PMC13314463; doi:10.3389/fmed.2026.1845205)
Supplement: Supplementary file 3 [file Data_Sheet_1.zip › 3.Beta_Diversity/NMDS/C372089/NMDS_binary_jaccard_2D.pdf]

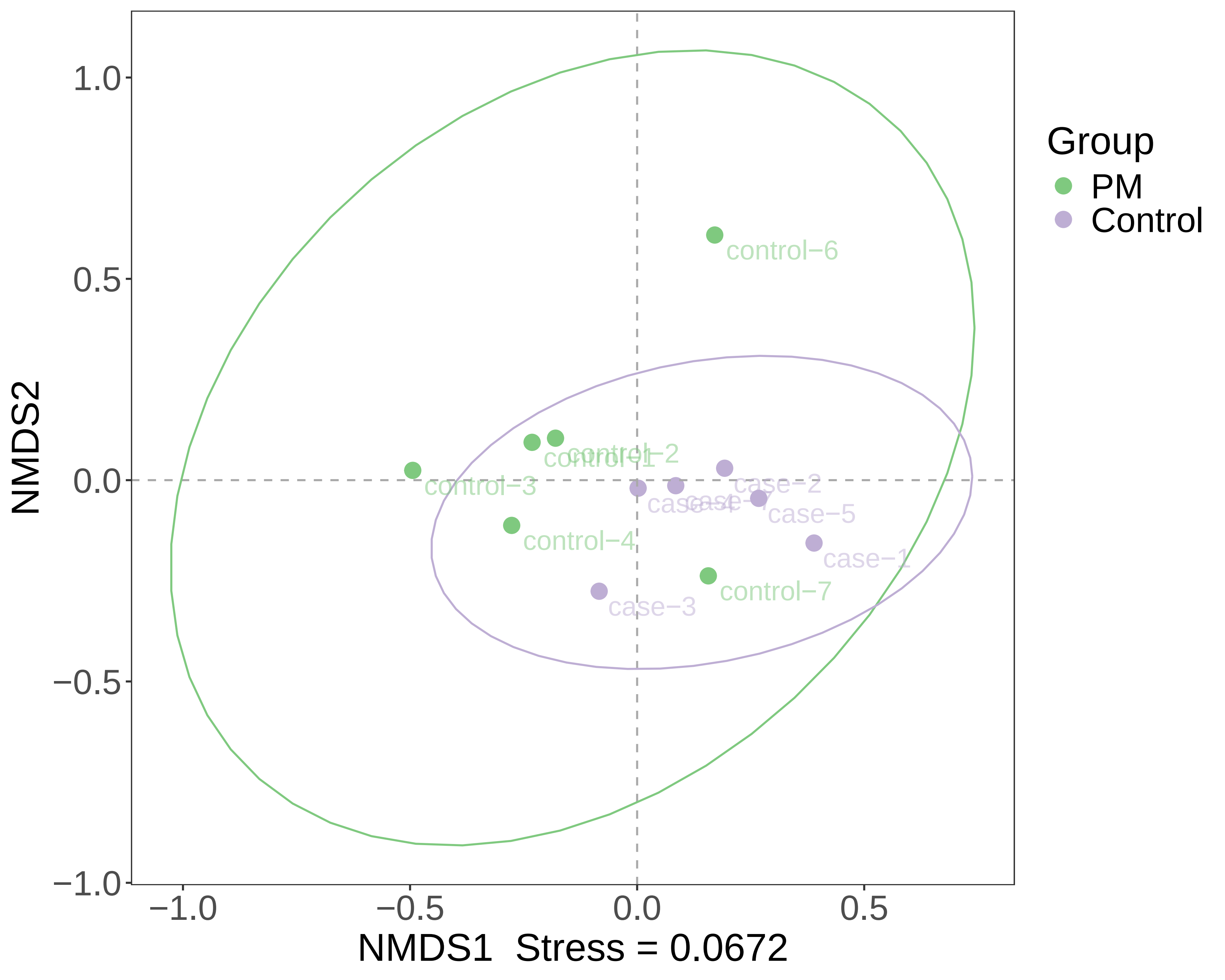

NMDS2
NMDS1 Stress = 0.0672
1.0
0.5
0.0
−0.5
−1.0
−1.0
−0.5
0.0
0.5
Group
PM
Control
control−6
control−2
control−1
control−3
control−4
control−7
case−2
case−4
case−7
case−5
case−1
case−3
